# K_V_11.1 Potassium Channel and the Na^+^/H^+^ Antiporter NHE1 Modulate Adhesion-Dependent Intracellular pH in Colorectal Cancer Cells

**DOI:** 10.3389/fphar.2020.00848

**Published:** 2020-06-10

**Authors:** Jessica Iorio, Claudia Duranti, Tiziano Lottini, Elena Lastraioli, Giacomo Bagni, Andrea Becchetti, Annarosa Arcangeli

**Affiliations:** ^1^Section of Internal Medicine, Department of Experimental and Clinical Medicine, University of Florence, Florence, Italy; ^2^Department of Biotechnology and Biosciences, University of Milano Bicocca, Milano, Italy

**Keywords:** hERG1, integrins, Collagen I, beta 1 integrin subunit, cariporide, lateral motility

## Abstract

Increasing evidence indicates that ion channels and transporters cooperate in regulating different aspects of tumor pathophysiology. In cancer cells, H^+^/HCO_3_^-^ transporters usually invert the transmembrane pH gradient typically observed in non-neoplastic cells, which is thought to contribute to cancer malignancy. To what extent the pH-regulating transporters are functionally linked to K^+^ channels, which are central regulators of cell membrane potential (V_m_), is unclear. We thus investigated in colorectal cancer cells the implication of the pH-regulating transporters and K_V_11.1 (also known as hERG1) in the pH modifications stimulated by integrin-dependent cell adhesion. Colorectal cancer cell lines (HCT 116 and HT 29) were seeded onto β1 integrin-dependent substrates, collagen I and fibronectin. This led to a transient cytoplasmic alkalinization, which peaked at 90 min of incubation, lasted approximately 180 min, and was inhibited by antibodies blocking the β1 integrin. The effect was sensitive to amiloride (10 µM) and cariporide (5 µM), suggesting that it was mainly caused by the activity of the Na^+^/H^+^ antiporter NHE1. Blocking K_V_11.1 with E4031 shows that channel activity contributed to modulate the β1 integrin-dependent pH_i_ increase. Interestingly, both NHE1 and K_V_11.1 modulated the colorectal cancer cell motility triggered by β1 integrin-dependent adhesion. Finally, the β1 integrin subunit, K_V_11.1 and NHE1 co-immunoprecipitated in colorectal cancer cells seeded onto Collagen I, suggesting the formation of a macromolecular complex following integrin-mediated adhesion. We conclude that the interaction between K_V_11.1, NHE1, and β1 integrin contributes to regulate colorectal cancer intracellular pH in relation to the tumor microenvironment, suggesting novel pharmacological targets to counteract pro-invasive and, hence, pro-metastatic behavior in colorectal cancer.

## Introduction

Ion channels and transporters are progressively emerging as pivotal modulators of different aspects of cancer cell behavior (Arcangeli et al., 2009; Lastraioli et al., 2015a). Such pleiotropic effects can be traced back to the regulation of either V_m_ (an effect mainly exerted by K^+^ channels; Huang and Jan, 2014), or of the concentration and intracellular distribution of specific ion species, such as Ca^2+^ (Bose et al., 2015) and H^+^ (Gadsby, 2009), or to the direct modulation of intracellular signaling pathways (Arcangeli et al., 2009; Becchetti et al., 2019). However, the molecular interactions between these mechanisms are poorly understood, and a unified picture of the cancer cell pathophysiology is still missing.

One of the K^+^ channels most often dysregulated in cancer is K_V_11.1 (or hERG1), which regulates the resting V_m_ in excitable cells (Bauer and Schwarz, 2018), as well as in cancers arising from excitable (e.g. neuroblastomas, Crociani et al., 2003) and non-excitable tissues. In particular, K_V_11.1 modulates intracellular signaling pathways triggered by integrin-mediated adhesion, both in leukemias (Pillozzi and Arcangeli, 2009) and solid cancers such as the colorectal (Crociani et al., 2013), pancreatic (Lastraioli et al., 2015b), gastric (Crociani et al., 2014) and mammary (Becchetti et al., 2017). The underlying mechanism involves the formation of a macromolecular complex between K_V_11.1 and β1-integrins, which promotes angiogenesis and triggers metastatic spread (Crociani et al., 2013; Becchetti et al., 2017). In pancreatic ductal adenocarcinoma cells, this occurs through the regulation of f-actin dynamics in filopodia (Manoli et al., 2019).

Cancer proliferation and migration are also controlled by intracellular pH, whose regulation is frequently dysregulated in tumors (Webb et al., 2011). Hence, targeting the pH regulating transporters has been suggested as a therapeutic strategy (Persi et al., 2018). Cancer cells generally display a higher activity of the V-type H^+^-ATPases expressed on the plasmalemma (Sennoune et al., 2004), the Na^+^/H^+^ exchanger NHE1 (Stock and Pedersen, 2017), the monocarboxylate transporters (MCTs) (Pinheiro et al., 2012), the Na^+^/HCO_3_^-^ cotransporters (NBCs) (Gorbatenko et al., 2014), and the membrane-associated carbonic anhydrases (CAs), such as CA II and CA IX (Mboge et al., 2018). The concerted activity of these proteins generally leads to an inverted transmembrane pH gradient, characterized by alkalization of intracellular pH (pH_i_) and extracellular acidosis. This is considered a hallmark of cancer metabolism (Sharma et al., 2015), being associated with increased tumor proliferation, invasion, metastasis, and thus higher aggressiveness and resistance to treatment (McCarty and Whitaker, 2010). Both the pH_i_ alkalinization and the extracellular acidosis sustained by the higher activity of H^+^ transporters in cancer cells promote cell proliferation, escape from apoptosis and metabolic adaptation (Webb et al., 2011). In addition, the inverted pH gradient is involved in the control of cell migration (Webb et al., 2011). An acid extracellular environment favors the formation of invadopodia and activates proteases that degrade the extracellular matrix, hence favoring cancer cell motility and invasiveness (Busco et al., 2010). Conversely, an alkaline pH_i_ stimulates cell motility by promoting cytoskeleton assembly and focal adhesion remodeling (Srivastava et al., 2008; Frantz et al., 2008), and hence is one of the main hallmarks of metastatic tumors (Stock and Schwab, 2009). In this scenario, a pivotal role is exerted by integrin receptors and by the Na^+^/H^+^ antiporter NHE1, whose reciprocal regulatory interaction was discovered in the late eighties (Schwartz et al., 1989; Demaurex et al., 1996). In particular, NHE1 is stimulated by cell adhesion, and in turn regulates cell attachment and spreading onto fibronectin (Harguindey et al., 2005; Stock and Schwab, 2006). In migrating cells, the production of a pH_i_ gradient along the axis of movement accompanies the accumulation of NHE1 at the migrating front, which localizes close to integrins (Grinstein et al., 1993; Plopper et al., 1995; Ludwig et al., 2013). The acidic pericellular environment at the leading edge is thought to increase the pH_e_-dependent avidity of integrins, which facilitates cell-matrix interactions and modulates adhesion strength (Lehenkari and Horton, 1999; Eble and Tuckwell, 2003; Stock et al., 2005; Stock et al., 2008). The corresponding local pH_i_ increase stimulates the focal adhesion dynamics. First, it supports the F-actin severing activity of cofilin (Frantz et al., 2008), which produces free-barbed-end actin in the lamellipodium. Second, it reduces the affinity of talin for actin (Srivastava et al., 2008). It is thus clear that the pH regulating mechanisms are essential determinants of the tumor microenvironment and the cancer cell crosstalk.

Based on the above premises, we investigated whether the pH-regulating transporters are functionally linked to K_V_11.1 channels, which are strongly dysregulated in cancer cells, and whose activity is tightly related to integrin receptors in modulating cancer cell proliferation and migration (Becchetti et al., 2019). In particular, we studied if pH regulating mechanisms provide a direct link between integrin-mediated hERG1-dependent cell adhesion and the tumor microenvironment. As a model, we used ColoRectal Cancer (CRC) cells, in which knowledge about K_V_11.1 physiology is particularly extensive.

## Materials and Methods

Unless otherwise indicated, chemicals and drugs were purchased from Sigma-Aldrich (St. Louis, USA).

### Cell Lines and Cell Culture

The human colon carcinoma cell lines HCT 116 and HT 29 were cultured at 37°C and 5% CO_2_ in air, in Roswell Park Memorial Institute (RPMI) 1640 Medium, with sodium bicarbonate (2 g/L) and 2mmol/L L-glutamine (“culture medium”), supplemented with 10% fetal bovine serum (FBS) (Euroclone, Italy). In all experiments, cells were starved overnight in culture medium without serum (“no-serum medium”) and detached, prior to experiment, with PBS plus 5 mM EDTA.

### Coating of Culture Substrates

Extracellular Matrix (ECM) proteins at the final amount per cm2 of surface area shown in brackets: Fibronectin (FN, 5 μg), Collagen-1 [Col-1, 10 μg; produced as reported in Hallowes et al. (1980)], Vitronectin [VN, 0.5 μg; produced as reported in Yatohgo et al. (1988)]. Coating with Polylysine (PL, 0.1 µg) was taken as a control of integrin-independent adhesion, while seeding onto uncoated dishes was our “no-adhesion” control. FN and VN were diluted in PBS, Col-1 in serum-free media, PL in bi-distilled water and plated to cover the entire growth surface, followed by 1 h incubation at either 37°C (for Col-1) or room temperature (for FN, VN and PL). After the coating procedure, incubation with Bovine Serum Albumin (BSA) for 15 min at 37°C was performed to block all the uncovered plastic sites.

### Measurement of pH_i_

To determine pH_i_, we used 2′,7′-Bis (2-carboxyethyl)-5 (6)-carboxyfluorescein acetoxymethyl esther (BCECF-AM). Cells were starved and detached as described above, and seeded (5 x 10^4^ cells/well) in no-serum medium onto uncoated or coated (see above) 96-well plates (clear bottom 96-well plate, polystyrene, TC-treated, clear flat bottom wells, sterile, w/lid, black; Corning, New York, USA). Cells were then incubated at 37°C in 5% CO_2_ for different times, in the absence or presence of different drugs (see below). At selected time points, the medium was removed and BCECF-AM (1μM ﬁnal concentration in loading solution (HBSS 1X plus 0.01% NaHCO_3,_ pH 7)) was added for 30 min at 37°C and 5% CO_2_. After incubation, cells were washed twice with loading solution at room temperature. For measurement of initial (time 0) pH_i_, cells were detached, kept in suspension in a 1.5 ml tube and incubated in BCECF-AM-containing solution for 30 min at 37°C. Next, they were washed twice with loading solution, poured in a 96-well plate at 5x10^4^ cells/well at room temperature, and immediately transferred to the microplate reader. Fluorescence intensity was immediately measured with a microplate reader (Infinite 200 PRO, Tecan, Switzerland) set at the following wavelengths: 440–490nm for excitation and 535 nm for emission. A calibration curve was set up using a high K^+^/Nigericin solution (135 mM KCl, 2 mM K_2_HPO_4_, 20 mM HEPES, 1.2 mM CaCl_2_ and 0.8 mM MgSO_4_), in a range of pH from 5.0 to 8.5. All pH values were calculated using 490/440nm ﬂuorescence ratio and applying standard curve and linear equations, as detailed in Grant and Acosta, 1997.

### Modulators of β1 Integrin-Mediated Adhesion

The mouse monoclonal anti-β1 integrin antibody BV7 (anti-β1 Ab, kindly gifted by Prof. P. Defilippi, University of Turin, Italy) (Martìn-Padura et al., 1994) was used to block β1-integrins as described in Hofmann et al. (2001). Briefly, cells were seeded onto Col-I in no serum medium, containing anti-β1 Ab (20 μg/ml), for 90 min at 37°C and 5% CO_2._ Cells (mostly detached) were collected, and pH_i_ mesaurement was performed as above described for time zero condition.

### Modulators of pH-Regulating Transporters and K_V_11.1

We used the following compounds: 100 µM acetazolamide (CA inhibitor; Parkkila et al., 2000), 10 µM amiloride (inhibitor of NHE1 and epithelial Na^+^ channel, ENaC; Masereel et al., 2003), 5 µM cariporide (specific NHE1 inhibitor; Hulikova et al., 2013), 30 µM S0859 (NBC inhibitor; Hulikova et al., 2013), and 40 µM E4031 (Tocris, Bristol, UK; K_V_11.1 blocker; Masi et al., 2005),

Drugs were added to the cells seeded on uncoated or coated surfaces at different time points. Preliminarily, all compounds were tested for their potential cytotoxic effects at all the used concentrations, by measuring cell viability with the trypan blue test (Pillozzi et al., 2018), at 30, 90 and 180 min of incubation. None of the modulators had any cytotoxic effect (**Table 1**).

**Table 1:** Percentage of alive cells after ICT modulators treatment for 30, 90, and 180 min (± s.e.m).

	30′		90′		180′	
	HCT 116	HT 29	HCT 116	HT 29	HCT 116	HT 29
**Acetazolamide**	99.01 ± 0.004	99.02 ± 0.004	98.02 ± 0.003	99.01 ± 0.009	97.02 ± 0.004	96.03 ± 0.004
**Amiloride**	99.02. ± 0.002	98.01 ± 0.008	99.03 ± 0.002	99.01 ± 0.002	98.02 ± 0.002	98.01 ± 0.004
**Cariporide**	98.01 ± 0.001	98.02 ± 0.001	99.01 ± 0.001	98.01 ± 0.001	98.03 ± 0.015	98.01 ± 0.001
**S0859**	99.02 ± 0.004	99.01 ± 0.004	98.01 ± 0.003	99.03 ± 0.009	97.01 ± 0.004	96.02 ± 0.004
**E4031**	99.01 ± 0.005	99.04 ± 0.003	99.01 ± 0.004	99.02 ± 0.005	99.01 ± 0.005	99.03 ± 0.007

### Lateral Motility Assay

Lateral motility was determined using 35 mm dishes and drawing 15 horizontal lines and 3 perpendicular lines on the dish bottom to generate a grid system. Plates were coated with Col-I and 5x10^5^ cells were seeded and allowed to attach for 90 min. Three wounds were drawn following the 3 horizontal lines. Subsequently, the following treatments were performed with drugs diluted in RPMI medium: control, E4031 40 µM, E4031 40 µM + cariporide 5 µM. Then, the distances between cells were measured at each mark point (where the 3 horizontal lines crossed the 15 vertical lines) using a light microscope. The widths measured at time 0 correspond to the W_0_ parameter. These different 45 points were measured again after 90′. Motility Index (MI) was assessed using the following formula: MI = 1 – W_t_/W_0_, where W_t_ is the width of the wounds after 90′.

Each treatment was performed in triplicate and the experiments were carried out at least 3 times.

### Co-Immunoprecipitation Experiments

For (co)-immunoprecipitation experiments cells were seeded and incubated on Col-I. Cells were gently collected by mild scraping and resuspended in ice cold PBS. Protein extraction, quantification and total lysate incubation with protein A/G agarose beads (Santa Cruz Biotechnology, Texas, USA) were performed as previously reported (Becchetti et al., 2017). In particular, the composition of the lysis buffer was the following: NP40 (150 mM), NaCl (150 mM), Tris-HCl pH 8 (50 mM), EDTA pH 8 (5 mM), NaF (10 mM), Na_4_P_2_O_7__(_10 mM), Na_3_VO_4_ (0.4 mM), and protease inhibitor cocktail (cOmplete Mini-Roche, Germany).

The following antibodies were used at the concentration of 5µg per mg of extracted proteins: LEAF Purified anti-human, Clone TS2/16 (BioLegend, California, USA) to immunoprecipitate the β1-integrin; mAb K_V_11.1 (MCK Therapeutics, Italy) to immunoprecipitate K_V_11.1. After overnight incubation, the immuno-complex was captured by adding 30 µl of protein A/G agarose beads for 2 h at 4°C (with rolling agitation). The agarose beads were washed 3 times in ice-cold wash buffer and 3 times in ice cold PBS followed by addition of 2X Laemli buffer (10 µl) and boiled for 5 min at 95°C. Afterwards, SDS-PAGE was performed. After electrophoresis, proteins were transferred onto PVDF membrane (previously activated) in blotting buffer under cold condition for 1 h at 100 V. The PVDF membrane was then blocked with 5% BSA in T-PBS (0.1% tween) solution for 3h at room temperature to cover the unspecific antibody binding sites on the membrane. SDS-PAGE and antibody incubation were performed as previously described (Becchetti et al., 2017). The following antibodies were used: anti β1-integrin, RM-12, polyclonal rabbit antibody, dilution 1:1,000 (Immunological Science, Italy); anti-Kv11.1, C54 polyclonal rabbit antibody, dilution 1:1,000 (DI.V.A.L TOSCANA S.R.L., Italy); anti-NHE1 polyclonal rabbit antibody, dilution 1:500 (Novus Biologicals, Colorado, USA) and anti-tubulin, monoclonal mouse, dilution 1:500 (Santa Cruz Biotechnology, Texas, USA). The following day the membrane was washed with T-PBS (0.1% tween) (15 min x 3 times) and appropriate secondary antibody: (i) conjugated with peroxidase enzyme was dissolved in 5% BSA in T-PBS (0.1% tween) (dilution 1:10.000) for at least 45 min and washed (15 min x 3 times), revealing was performed using ECL solution in dark room (anti-C54 primary antibody) and (ii) for all other primary antibodies, IRDYe 800 CW (LI-COR Biosciences, Nebraska, USA) was dissolved in 5% BSA in T-PBS (0.1% tween) (dilution 1:20.000) for at least 45 min and washed (15 min x 3 times) before membrane scanning using LI-COR Odyssey Scanner (Biosciences, Nebraska, USA).

### Protein Quantification

Data were analyzed with ImageJ and graphs were plotted with OriginPro8. When quantifying variations in K_V_11.1-β1 integrin interaction, the signal for co-immunoprecipitated protein was first divided by the signal of the protein used for immunoprecipitation and then normalized to the signal of the corresponding protein in the total lysate.

### Statistical Analysis

OriginPro8 was used for analysis. Data groups were tested for normality (Shapiro-Wilk test) and variance homogeneity (Welch test). Statistical significance for two sample analysis was carried out with unpaired t-test. Multiple comparisons were carried out by One-way ANOVA, with post-hoc Bonferroni test. A p value ≤ 0.05 was considered statistically significant.

## Results

### Cell Adhesion Mediated by β1 Integrin Produces an Early pH_i_ Alkalinization in HCT 116 and HT 29 CRC Cells

**Figure 1:**
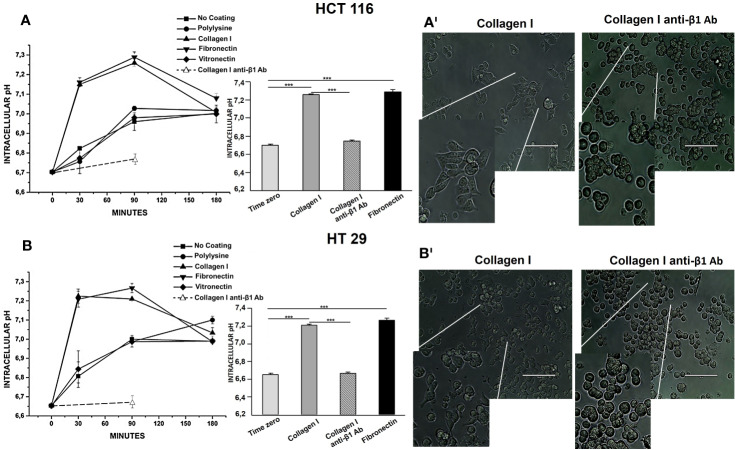
Effect of Collagen I, Fibronectin, and Vitronectin on pH_i_ in HCT 116 and HT 29 cells. The time course of pH_i_ is reported in panel **(A)** (HCT 116) and in panel **(B)** (HT 29). Simbol ▪: No coating surface, •: PL coating, ▲: Col-I coating, ▼: FN coating, ♦: VN coating, ○: Col-I anti-β1 Ab. On the right of the panel, 90 min pH_i_ values are reported. Light grey bar: No coating surface, dark grey bar: Col-I, black bar: FN and striped bar: Col-I anti-β1 Ab. Number represent mean ± s.e.m (of three different experiments). *, *P* < 0.05; **, *P* < 0.01 and ***, *P* < 0.001. *p value after 30 min of seeding, panel* **(A)**: ***P < 0.001: Col-I vs Control: 0.0005, FN vs Control: 0.0005; *p value after 90 min of seeding, panel* **(A)**: ***P < 0.001: Col-I vs Control: 0.0001, FN vs Control: 0.0001; *P < 0.05: No coat vs Control: 0.01, PL vs Control: 0.01 and VN vs Control: 0.02*. p value after 180 min of seeding, panel* **(A)**: *P < 0.05: No coat vs Control: 0.01, PL vs Control: 0.01 and VN vs Control: 0.01, Col-I vs Control: 0.01, FN vs Control: 0.01. *p value after 30 min of seeding, panel* **(B)** ***P < 0.001: Col-I vs Control: 0.0001 and FN vs Control: 0.0001; *p value after 90 min of seeding, panel* **(B)** ***P < 0.001: Col-I vs Control: 0.0001 and FN vs Control: 0.0001; *P < 0.05: No coat vs Control: 0.01, PL vs Control: 0.01 and VN vs Control: 0.01*. p value after 180 min of seeding, panel* **(B)** *P < 0.05: No coat vs Control: 0.01, PL vs Control: 0.01, VN vs Control: 0.01, Col-I vs Control: 0.01 and FN vs Control: 0.01. *p value after 30 min of seeding, panel* **(A)**: ***P < 0.001: Col-I vs No coat: 0.001 and FN vs No coat: 0.001*. p value after 90 min of seeding, panel* **(A)**: Col-I vs No coat: 0.0001, FN vs No coat: 0.0001; Col-I anti-β1 Ab vs Col-I: 0.0007; Col-I anti-β1 Ab vs FN: 0.0006. *p value after 30 min of seeding, panel* **(B)** ***P < 0.001: Col-I vs No coat: 0.001 and FN vs No coat: 0.001*. p value after 90 min of seeding, panel* **(B)** Col-I vs No coat: 0.001 and FN vs No coat: 0.001. Col-I anti-β1 Ab vs Col-I: 0.0006; Col-I anti-β1 Ab vs FN: 0.0005. Representative images of cells seeded for 90 min on Col-I, no treated and treated with anti-β1 Ab are reported in panels A^I^ (HCT 116) and B^I^ (HT 29), 100 µm scale bar. The conditions are shown on the top of each picture. Cells seeded on Col-I are elongated and attached, cells seeded on Col-I and treated with anti-β1 Ab are round and detached.

HCT 116 and HT 29 cells were incubated in serum-free medium for different times (0–180 min) onto different ECM substrates: Col-I, FN, and VN. Polylysine or no-coated plastic surfaces were used as integrin-independent or “no-adhesion” controls, respectively. At different time points, pH_i_ was determined by BCECF-AM. Although with slightly different time courses, both cell lines underwent an early pH_i_ increase between 0 and 90 min (**Figures 1A, B**). At 90 min of incubation, pH_i_ was significantly higher in cells seeded onto Col-I and FN, i.e., two substrates recognized by β1 integrins, which are well expressed in both cell lines (**Table 2**). Indeed, treatment with a β1 integrin blocking antibody (BV/, Martìn-Padura et al., 1994) (indicated as “anti-β1 Ab” in **Figure 1**) not only blocked cell adhesion (panel A’ and B’), but also prevented the pH_i_ increase triggered by cell adhesion onto Col-I. In particular, pH_i_ increased from 6.7 at time 0 (i.e., before seeding) to 7.2 at 90 min in cells seeded either onto Col-I or FN. In contrast, pH_i_ remained close to the time 0 value (6.74±0.006 for HCT 116 and 6.66±0.014 for HT 29) in CRC cells seeded onto Col-I and treated with the anti-β1 Ab (insets to **Figures 1A, B**). The pH_i_ alkalinization was much smaller in cells seeded onto VN (in agreement with the very low expression of β3-integrins in both cell lines, see **Table 2**), or polylysine or no-coating conditions. Subsequently (i.e., after 90 min of incubation), the pH_i_ observed in cells seeded on Col-I and FN progressively returned to the control value. At 180 min, cells displayed a pH_i_ around 7.0, irrespective of growing conditions (**Figures 1A, B**). Such values were maintained for at least 24 h (the complete data set is given in **Table 3**). We conclude that the β1 integrin-mediated adhesion triggers an early and transient pH_i_ alkalinization from 6.7 to 7.2 in CRC cells.

**Table 2:** Integrin profile and ICT expression of HCT 116 and HT 29 cell lines.

	HCT 116	HT 29
**α_1_β_1(Col-I)_, α_2_β_1(Col-I)_**	+ ^(Boudjadi et al., 2016;^ ^Gout et al., 2001)^	+ ^(Boudjadi et al., 2016;^ ^Pelillo et al., 2015)^
**α_3_β_1(FN),_ α_4_β_1(FN)_**	+ ^(Pelillo et al., 2015;^ ^Ito et al., 2004)^	+ ^(Gout et al., 2001;^ ^Ito et al., 2004)^
**α_5_β_1(FN)_**	+ ^(Pelillo et al., 2015)^	- ^(Schmidt et al., 1998)^
**α_8_β_1(FN)_**	- ^(Benoit et al., 2010)^	- ^(Benoit et al., 2010)^
**α_V_β_3(FN),_ α_V_β_3(VN)_**	- ^(Pelillo et al., 2015)^	- ^(Christenheit et al., 2016)^
**α_V_β_6(FN)_**	+ ^(Kim et al., 2018)^	+ ^(Kim et al., 2018)^
**Kv 11.1**	+ ^(Pillozzi et al., 2018)^	+ ^(Pillozzi et al., 2018)^
**CA IX**	- ^(McIntyre et al., 2012)^	+ ^(McIntyre et al., 2012)^
**NHE1**	+ ^(Hulikova et al., 2011)^	+ ^(Hulikova et al., 2011)^
**NBCe1**	+ ^(Hulikova et al., 2011)^	+ ^(Hulikova et al., 2011)^
**MCT1**	+ ^(Jin et al., 2019)^	+ ^(Jin et al., 2019)^
**MCT4**	+ ^(Choi et al., 2019)^	+ ^(Choi et al., 2019)^

**Table 3:** Complete set of raw pH_i_ values.

HCT 116	0’	30’	90’	180’	360’	1440
**No Coating**	6.70 ± 0.008	6.82 ± 0.005	6.96 ± 0.045	7.00 ± 0.003	7.10 ± 0.004	7.12 ± 0.013
**Polylysine**	6.70 ± 0.008	6.75 ± 0.036	7.03 ± 0.011	7.02 ± 0.012	7.12 ± 0.013	7.12 ± 0.012
**Collagen I**	6.70 ± 0.008	7.15 ± 0.009	7.26 ± 0.007	7.01 ± 0.005	7.11 ± 0.008	7.01 ± 0.015
**Fibronectin**	6.70 ± 0.008	7.16 ± 0.023	7.29 ± 0.026	7.08 ± 0.004	7.05 ± 0.014	7.18 ± 0.04
**Vitronectin**	6.70 ± 0.008	6.77 ± 0.011	6.98 ± 0.016	6.99 ± 0.045	7.00 ± 0.025	7.10 ± 0.015
**HT 29**	**0’**	**30’**	**90’**	**180’**	**360’**	**1440**
**No Coating**	6.65 ± 0.014	6.81±0.035	7.00 ± 0.021	6.99 ± 0.01	7.09 ± 0.01	7.09 ± 0.01
**Polylysine**	6.65 ± 0.014	6.84 ± 0.096	6.99 ± 0.006	7.12 ± 0.02	7.16 ± 0.001	7.16 ± 0.001
**Collagen I**	6.65 ± 0.014	7.22 ± 0.037	7.21 ± 0.005	7.03 ± 0.027	7.11 ± 0.023	7.11 ± 0.023
**Fibronectin**	6.65 ± 0.014	7.21 ± 0.042	7.26 ± 0.024	6.98 ± 0.006	7.03 ± 0.028	7.03 ± 0.028
**Vitronectin**	6.65 ± 0.014	6.84 ± 0.037	6.98 ± 0.027	6.99 ± 0.011	7.01 ± 0.017	7.01 ± 0.017

### The pH_i_ Variations Triggered by β1 Integrin-Dependent Adhesion in CRC Cells Depend on NHE1 Activation and Are Modulated by K_V_11.1 Activity

**Figure 2:**
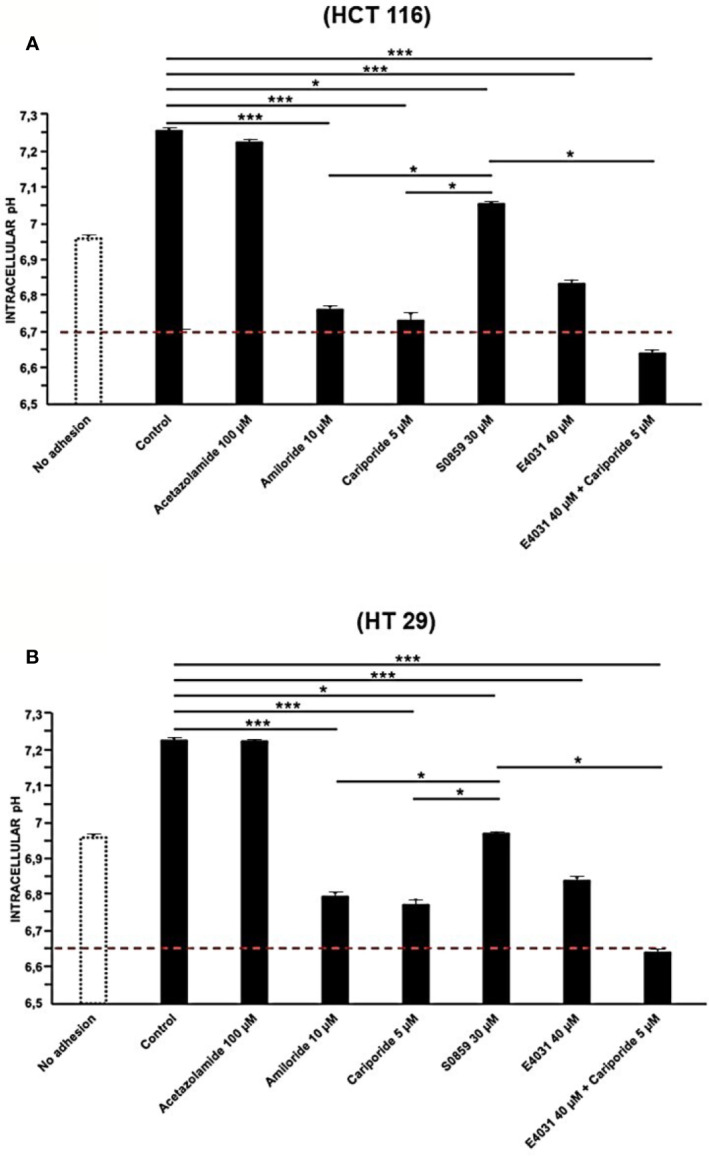
Effect of Acetazolamide, Amiloride, S0859, Cariporide, E4031, and E4031 plus Cariporide on pH_i_ in cells seeded on collagen I, 90 min treatment, in HCT 116 and HT 29 cells. pH_i_ values of HCT 116 are reported in panel **(A)** and for HT 29 in panel **(B)**. Red line: pH_i_ value at time zero. Number represent mean ± s.e.m (of three different experiments). *, *P* < 0.05 and ***, *P* < 0.001. *p value panel* **(A)**: ***P < 0.001: Control vs Amil: 1.9e^^-05^, Control vs Carip: 1.9e^^-05^, Control vs E4031: 0.0001, Control vs E4031+Carip: 1.8e^^-05^Acet vs Carip: 1.7e^^-05^; *P < 0.05: Control vs S0859: 0.03, Acet vs S0859: 0.03, Amil vs S0859: 0.02; Carip vs S0859: 0.02; S0859 vs E4031+ Carip: 0.02. *p value panel* **(B)**: ***P < 0.001: Control vs Amil: 1.5e^^-05^, Control vs Carip: 1.4e^^-05^, Acet vs Carip: 1.3e^^-05^; Control vs E4031: 0.0001 and Control vs E4031+Carip: 1.9e^^-05^; *P < 0.05: Control vs S0859: 0.04, Acet vs S0859: 0.03; S0859 vs E4031+ Carip: 0.02.

To better determine the mechanism of integrin-dependent pH_i_ increase, we applied blockers of the different pH-regulating transporters expressed in CRC cells (**Table 2**). In particular, we tested acetazolamide (a wide CA inhibitor), amiloride (an NHE blocker, in particular of NHE1, as well as of ENaC), cariporide (a specific NHE1 inhibitor), and S0859 (an inhibitor of all NBCs). Drugs were used at the concentrations indicated in Materials and Methods, on cells seeded onto Col-I for 90 min, since the beginning of the experiment. Acetazolamide had no effect on pH_i_ of either cell line, whereas both amiloride and cariporide produced a statistically significant decrease of pH_i_ which reached values comparable to those detected in cells before seeding (dotted line in **Figure 2**). The same effect was produced by cariporide. The treatment with S0859 produced a reduction of pH_i_, although much lower compared to that obtained with amiloride (**Figures 2A, B**).

These results suggest that the early alkalinization triggered by β1 integrin-mediated adhesion is mostly sustained by the activity of the Na^+^/H^+^ antiporter NHE1, with a lesser contribution of NBC, and scarse involvement of carbonic anhydrases.

We then tested whether K_V_11.1 was involved in the integrin-dependent pH_i_ alkalinization. To this purpose, cells were treated with the K_V_11.1 blocker E4031, at 40 µM (Masi et al., 2005). After 90 min of cell adhesion on Col-I, both HCT 116 and HT 29 cells treated with E4031 showed pH_i_ values significantly more acidic compared to the untreated controls (**Figures 2A, B**). Hence, the activity of K_V_11.1 appears to control NHE1 activation, after β1 integrin-mediated adhesion. This interpretation was supported by the observation that the combined treatment with E4031 and cariporide had no further effect on the pH_i_ value obtained after NHE1 inhibition by cariporide (**Figures 2A, B**).

### Blockade of Either NHE1 or K_V_11.1 Inhibits Lateral Motility of CRC Cells

**Figure 3:**
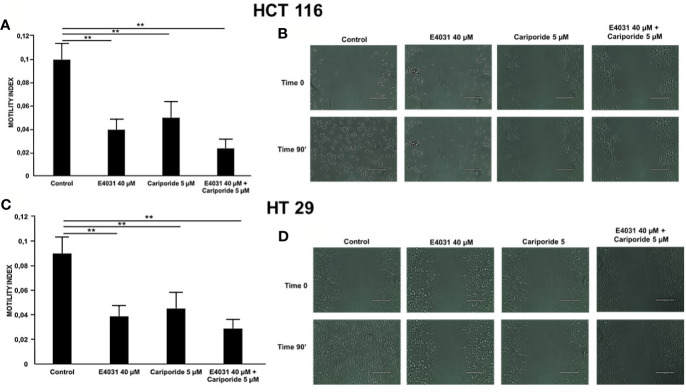
Effect of E4031 and E4031 plus Cariporide on motility index in cells seeded on col-I, 90 min treatment. Motility index values are reported for HCT 116 in panel **(A)** and for HT 29 in panel C. Number represent mean ± s.e.m (of three different experiments). Representative images are reported in panel **(B)** for HCT 116 and in panel **(D)** for HT 29, 200 µm scale bar. In figure the statistically significant differences between control and treatments are reported. **, *P* < 0.01. *p value panel* **(A)**: **, *P* < 0.01: Control vs E4031: 0.002 and Control vs E4031+Carip: 0.001. *p value panel* **(C)**: **, *P* < 0.01: Control vs E4031: 0.003 and Control vs E4031+Carip: 0.001.

Next, based on the known correlation between pH_i_ and cell motility, we performed experiments of lateral motility on our CRC cell lines, which were seeded onto Col-I for 90 min, and treated with either E4031, or cariporide, or a combination of both. Both cariporide and E4031 produced a statistically significant reduction of the motility index compared to untreated cells (**Figures 3A, B**). The combined treatment with E4031 and cariporide only slightly increased the inhibitory effects of the single treatments on the motility index of either cell line. We conclude that both K_V_11.1 and NHE1 are involved in controlling the β1 integrin-dependent cell motility in CRC cells.

### β1-Integrin, K_V_11.1, and NHE1 Form a Macromolecular Complex

**Figure 4:**
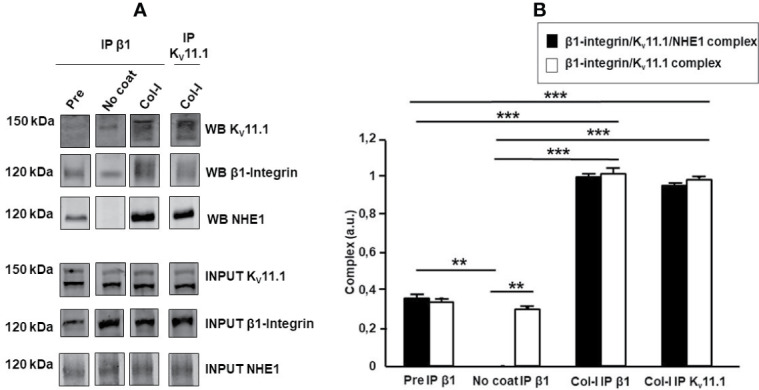
β1-integrin, K_V_11.1, and NHE1 protein complex. **(A)** Co-immunoprecipitation of β1 Integrin, Kv 11.1 and NHE1 in HCT 116 cells, seeded on no coating surface and Col-I for 90 min. Densitometric analysis is reported in panel **(A)**. In panel A with “WB” is indicated the protein signal in the co-ip and with “INPUT” the protein signal in the total lysate. Pre-seeding condition is reported as pre, No coating as No coat and Collagen I as Col-I; The immunoprecipitation with anti β1 integrin antibody is indicated as IP β1 and with anti K_V_11.1 antibody is reported as IP K_V_11.1. Complex quantification is reported in panel B, black bar: β1-integrin, K_V_11.1 and NHE1 protein complex and white bar: β1-integrin and K_V_11.1 protein complex. Number represent mean ± s.e.m (of three different experiments). **, *P* < 0.01 and ***, *P* < 0.001. *p value panel* **(B)**, ***, *P* < 0.001: β1-integrin/K_V_11.1/NHE1 complex, Pre vs Col-I IP β1, p: 0.0008; β1-integrin/K_V_11.1 complex, Pre vs Col-I IP β1, p: 0.00075; β1-integrin/K_V_11.1/NHE1 complex, Pre vs Col-I IP K_V_11.1, p: 0.00076; β1-integrin/K_V_11.1 complex, Pre vs Col-I IP K_V_11.1 p: 0.00074. β1-integrin/K_V_11.1/NHE1 complex, No coat vs Col-I IP β1, p: 1.7e^^-05^; β1-integrin/K_V_11.1 complex No coat vs Col-I IP β1, p: 0.00072; β1-integrin/K_V_11.1/NHE1 complex, No coat vs Col-I IP K_V_11.1, p: 1.9e^^-05^; β1-integrin/K_V_11.1 complex, No coat vs Col-I IP K_V_11.1, p: 0.00073. **, *P* < 0.01: β1-integrin/K_V_11.1/NHE1 complex, Pre vs No coat: 0.002; β1-integrin/K_V_11.1/NHE1 complex vs β1-integrin/K_V_11.1 complex, No coat IP β1: 0.002. Cropped images of blots are reported.

We previously showed that cell adhesion onto β1 integrin-dependent substrates (e.g., FN or Col-I), induces K_V_11.1 activation, as well as the formation of a macromolecular signaling complex between the channel and β1 integrin on the plasma membrane of HCT 116 cells (Crociani et al., 2013). We thus hypothesized that NHE1 could be also recruited in such complex, which could account for the functional cross-talk between integrin receptors, K_V_11.1, and NHE1 in CRC cells. Hence, we seeded HCT116 cells on either uncoated or Col-I-coated surfaces for 90 min, and immunoprecipitated the extracted proteins with anti-β1 integrin or anti-K_V_11.1 antibodies. Blots were then revealed, respectively, with anti-K_V_11.1 or anti-β1 integrin antibodies, as well as with anti-NHE1 antibodies. We observed that β1-integrin co-immunoprecipitated with both K_V_11.1 and NHE1 in CRC cells before cell seeding (“pre seeding” in **Figure 4**), indicating the formation of a β1/Kv11.1/NHE1 complex, whose assembly was further promoted by cell adhesion onto Col-I for 90 min (lanes 3 and 4 in **Figure 4**). On the contrary, in cells seeded onto uncoated surfaces, only a weak co-immunoprecipitation was observed between β1-integrin and K_V_11.1, and no association was observed with NHE1. We conclude that cell adhesion onto Col-I stimulates the formation of a macromolecular complex between β1-integrin, K_V_11.1, and NHE1.

## Discussion

In the present paper, we provide evidence that, in CRC cells, the β1 integrin-mediated adhesion onto ECM proteins such as Col-I and FN triggers an early and transient pH_i_ alkalinization, from 6.7 to 7.2. The effect is caused by NHE1 activation and is modulated by the activity of the voltage-dependent K^+^ channel K_V_11.1. The transporter and the channel appear to cooperate in sustaining the ECM-induced CRC cell motility. Their action is accompanied by the formation of a macromolecular complex between the β1 integrin, K_V_11.1 and NHE1.

The rapid β1 integrin-dependent pH_i_ alkalinization in CRC cells is similar to the one initially reported in bovine capillary endothelial cells (Schwartz et al., 1991), which is also induced by integrin engagement (mainly β1), and sustained by activation of the Na^+^/H^+^ exchanger. In our model, we confirmed the NHE1 involvement by showing that ECM-dependent alkalinization was blocked by cariporide.

Following Schwartz’s seminal observation, the pH regulatory role of NHE1 in normal and cancer cells has been receiving increasing attention (Stock and Pedersen, 2017). In particular, in CRC cells, both H^+^ extrusion through NHE1 and HCO_3_^-^ influx through NBCe1 give a significant contribution to pH_i_ regulation. However, while HCO_3_^-^ influx appears to represent a constitutive element of pH_i_ regulation, the NHE1-mediated H^+^ efflux may vary, depending on culture conditions, e.g. 2D vs 3D cultures (Hulikova et al., 2011). In CRC cells, we found that the Na^+^/HCO_3_^-^ cotransporter, although present, provides only a weak contribution to the integrin-dependent alkalinization in CRC cells. In fact, NHE1 appears to constitute the main molecular device linking the ECM microenvironment to pH_i_ regulation.

Numerous mechanisms leading to NHE1 activation have been described in the various cell types in which the transporter is expressed (Orlowski and Grinstein, 2004). Stimuli such as growth factors, peptide hormones etc., which activate receptor tyrosine kinases and G protein-coupled receptors, enhance NHE1 activity, through the involvement of the mitogen-activated, extracellular signal-related kinase (MEK-ERK)-p90rsk. The latter phosphorylates NHE1, and enables its binding to the multifunctional scaffolding protein 14-3-3, which in turn serves as a hub for the assembly of other signaling molecules which eventually enhance cation exchange (Orlowski and Grinstein, 2004). In this scenario, it is not surprising that integrins exert a stimulatory role on NHE1, as they are known to trigger intracellular signaling pathways that can lead to NHE1 activation (Putney et al., 2002). Integrins, and the tumor microenviroment as a whole, can contribute to trigger a complex signaling pathway which in turn regulates NHE1-dependent motility and invasion in different cancer cells (Cardone et al., 2005). In particular, NHE1 is linked to the actin cytoskeleton and integrates phosphorylation signals arising from kinases which are involved in cytoskeletal reorganization and cell motility. In addition, NHE1 is preferentially localized in pseudopodia, focal adhesion plates, and invadopodia in migrating cells (Paradiso et al., 2004; Patel and Barber, 2005; Clement et al., 2013). In this context, a slight alkalinization mediated by NHE1 was found to regulate the cofilin-mediated actin assembly (Frantz et al., 2008), a central mechanism in cell protrusion. Hence, NHE1 and cofilin respectively act as a pH regulator and a pH sensor, to mediate actin filament assembly.

The most novel result emerging from our data is that the K_V_11.1 channel is implicated in the pH_i_ alkalinization triggered by integrin-mediated cell adhesion to ECM proteins, and sustained by NHE1 activity. K_V_11.1 is over-expressed in many cancer types, including CRC (Lastraioli et al., 2004; Lastraioli et al., 2012; Crociani et al., 2013; Iorio et al., 2020). In cancers, β1 integrin-mediated adhesion to FN or Col-I activates K_V_11.1, and induces the formation of a macromolecular functional complex on the plasma membrane which comprises the channel and the integrin itself. This occurs preferentially when the channel is in the closed conformation, and leads to the activation of signaling pathways, which also involve the scaffold protein 14-3-3, and in turn control different aspects of cancer cell behavior (Becchetti et al., 2017; Becchetti et al., 2019). The recruitment of NHE1 in the K_V_11.1/β1 integrin complex could give rise to the formation of a signaling hub, facilitating NHE1 activation and hence a localized pH_i_ alkalinization, which in turn could affect the reorganization of actin filaments, presumably regulated by cofilin activation. This agrees with our recent observations in pancreatic ductal adenocarcinoma cells, where K_V_11.1 regulates cell migration through a reorganization of f-actin in stress fibers and a modulation of filopodia formation and dynamics (Manoli et al., 2019).

The identification of the signaling mechanisms underlying K_V_11.1 and NHE1 interaction triggered by β1 integrin–mediated adhesion needs further experiments. Nevertheless, the interplay between a K+ channel and the pH regulating transporter NHE1 that we describe in the present paper can be considered of relevance in the context of CRC invasiveness/motility. This aspect is often dependent on a complex interaction between cancer cells and the tumor microenvironment, and in particular with ECM proteins like collagens and fibronectin (Arcangeli, 2011). Finally, targeting the integrin/ion channel/NHE1 molecular hub might represent a therapeutic option to fight cancer invasiveness.

## Data Availability Statement

The datasets generated for this study are available on request to the corresponding author.

## Author Contributions

JI performed the experiments, analyzed the data, prepared the figures, wrote the manuscript. CD performed the experiments and wrote the manuscript. TL performed the experiments. EL helped in the analysis of the data and reviewed the manuscript. GB contributed to experiments and figures. AB reviewed manuscript. AA designed this project and wrote the manuscript.

## Funding

This work was supported by the Associazione Italiana per la Ricerca sul Cancro (AIRC, Grant N° IG 21510 and Grant N°IG 15627), by the European Union’s Horizon 2020 research and innovation programme under the Marie Skłodowska-Curie grant agreement No 813834, by PRIN 2017 (LIONESS) and by Doctorate Course in Genetics, Oncology and Clinical Medicine (Department of Medical Biotechnologies, University of Siena, Siena, Italy).

## Conflict of Interest

The authors declare that the research was conducted in the absence of any commercial or financial relationships that could be construed as a potential conflict of interest.
