# Supplementary material for: KV11.1 Potassium Channel and the Na+/H+ Antiporter NHE1 Modulate Adhesion-Dependent Intracellular pH in Colorectal Cancer Cells
Source: Front Pharmacol. 2020 Jun 10;11:848. doi: 10.3389/fphar.2020.00848 (PMC7297984; doi:10.3389/fphar.2020.00848)
Supplement: Supplementary file 1 [file DataSheet_1.zip › wb input original membrane_Iorio et al_18052020.pdf]

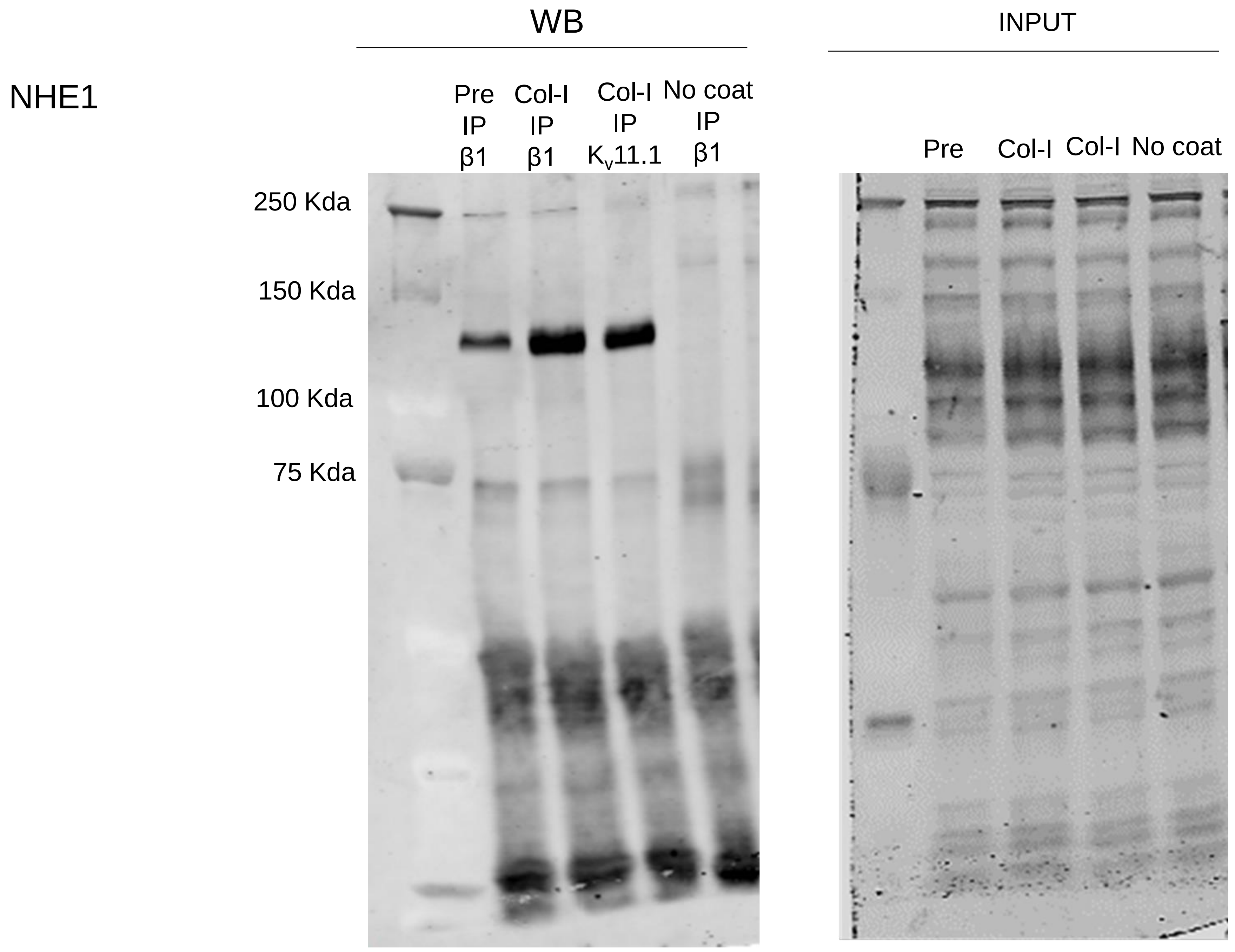
WB
INPUT
NHE1
Pre IP β1
Col-I IP β1
Col-I IP Kv11.1
No coat IP β1
250 Kda
150 Kda
100 Kda
75 Kda
Pre
Col-I
Col-I
No coat

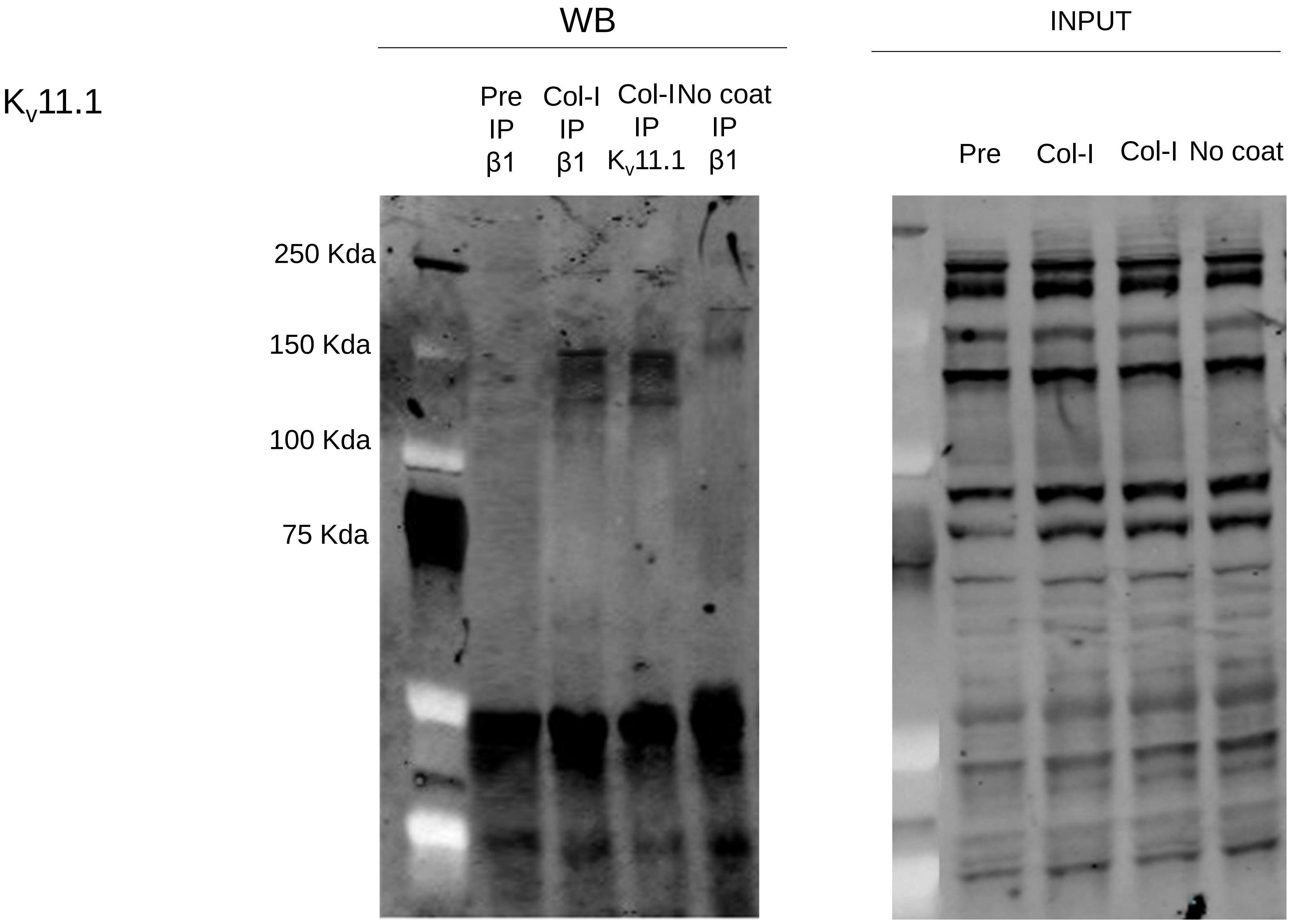
WB
INPUT
K_v11.1
Pre IP β1
Col-I IP β1
Col-I IP K_v11.1
No coat IP β1
Pre
Col-I
Col-I
No coat
250 Kda
150 Kda
100 Kda
75 Kda

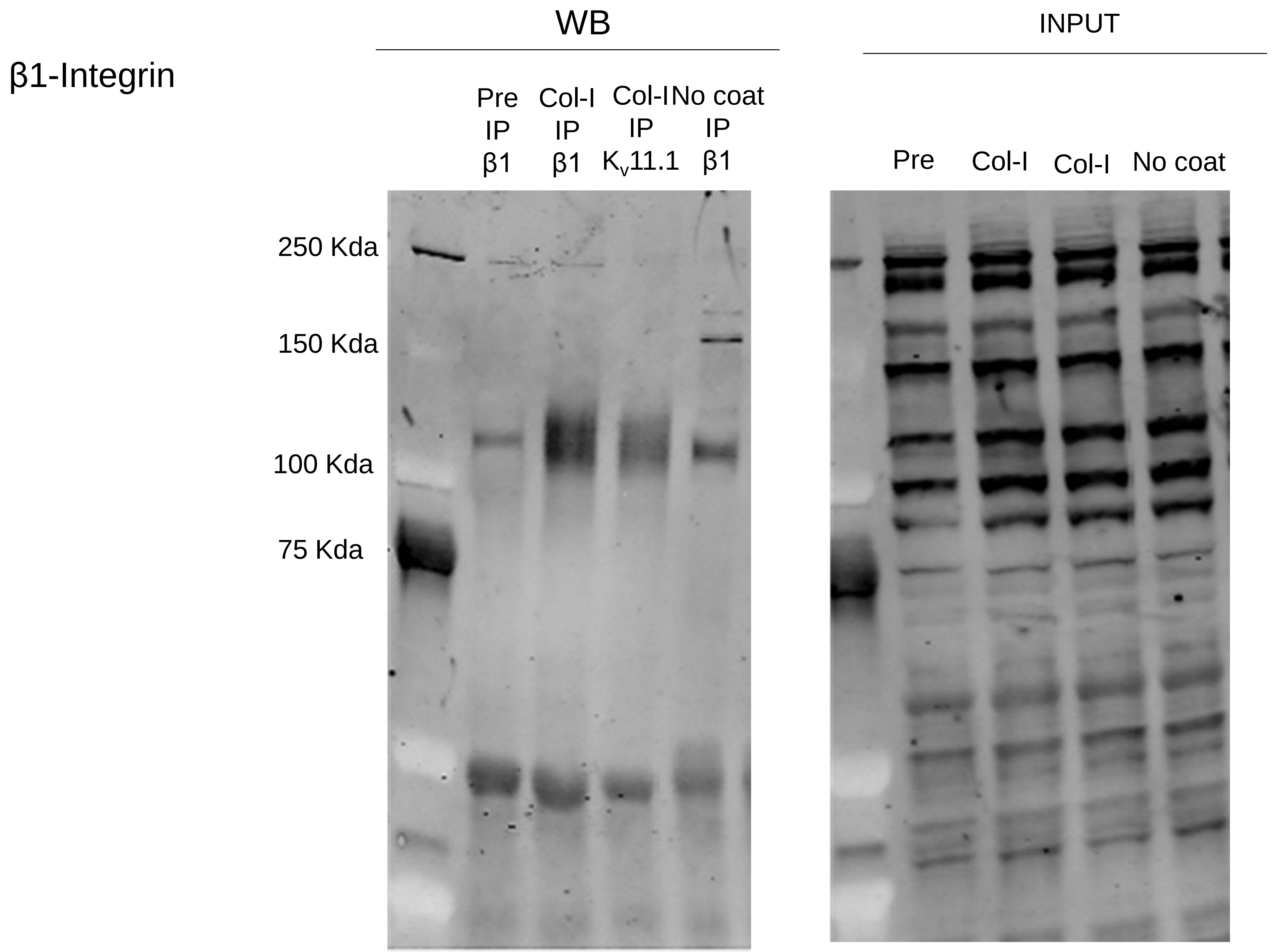

β1-Integrin
WB
INPUT
Pre IP β1
Col-I IP β1
Col-I IP $K_v11.1$
No coat IP β1
Pre
Col-I
Col-I
No coat
250 Kda
150 Kda
100 Kda
75 Kda
