# Supplementary figures and images for: KV11.1 Potassium Channel and the Na+/H+ Antiporter NHE1 Modulate Adhesion-Dependent Intracellular pH in Colorectal Cancer Cells
Source: Front Pharmacol. 2020 Jun 10;11:848. doi: 10.3389/fphar.2020.00848 (PMC7297984; doi:10.3389/fphar.2020.00848)

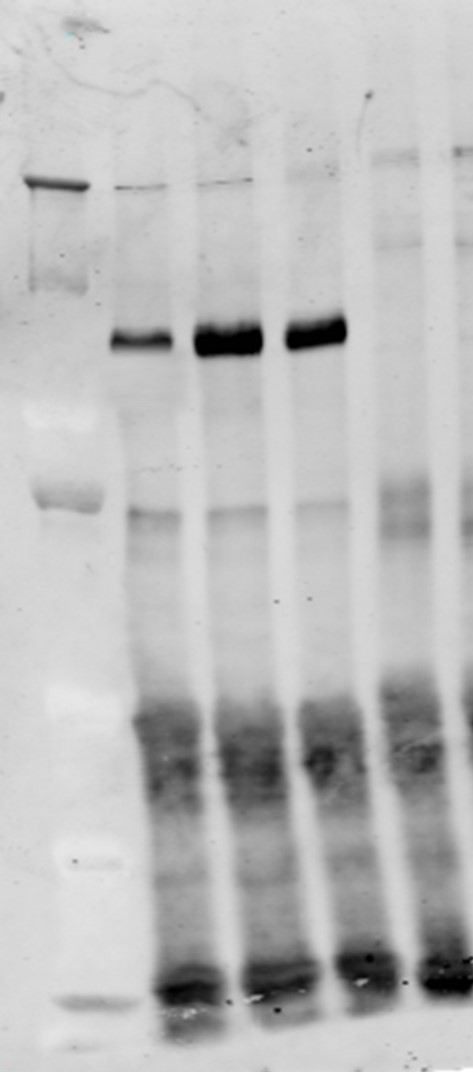

Supplement: Supplementary file 1 [file DataSheet_1.zip › nhe1 wb.jpg]

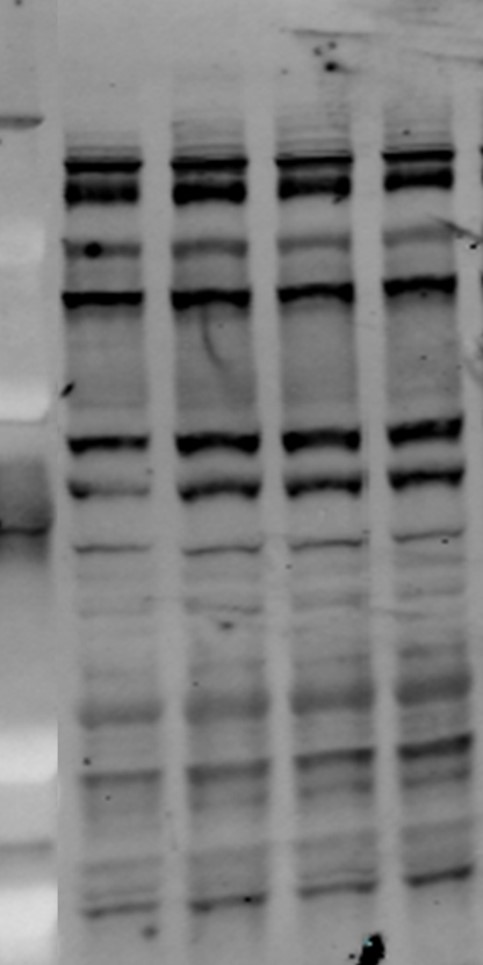

Supplement: Supplementary file 1 [file DataSheet_1.zip › herg1 input.jpg]

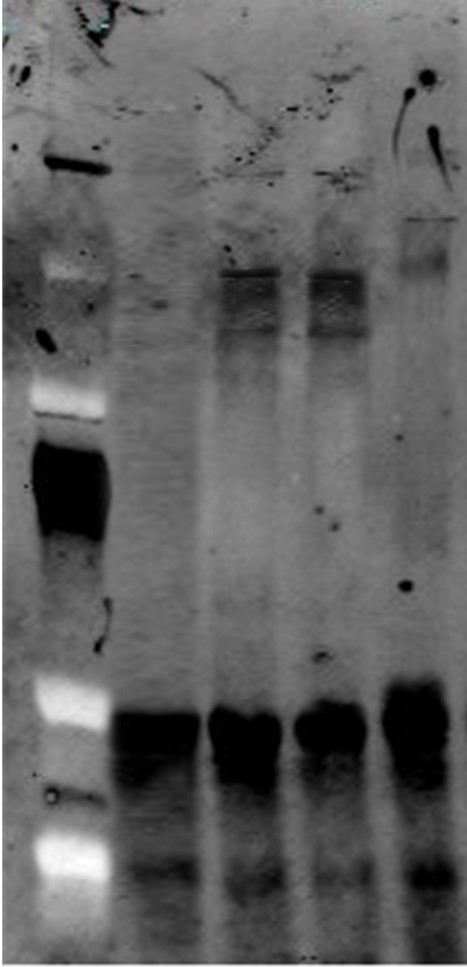

Supplement: Supplementary file 1 [file DataSheet_1.zip › hERG1 wb.jpg]

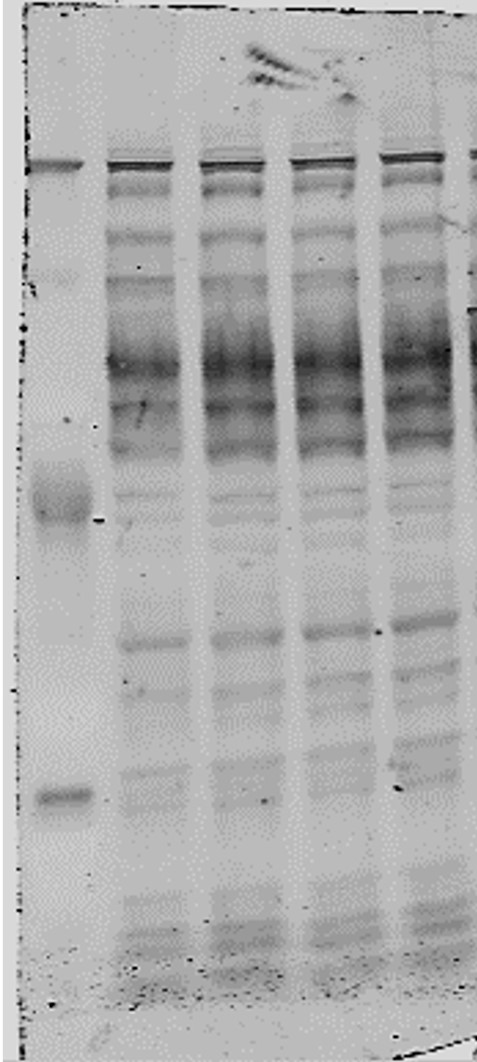

Supplement: Supplementary file 1 [file DataSheet_1.zip › nhe1 input.jpg]

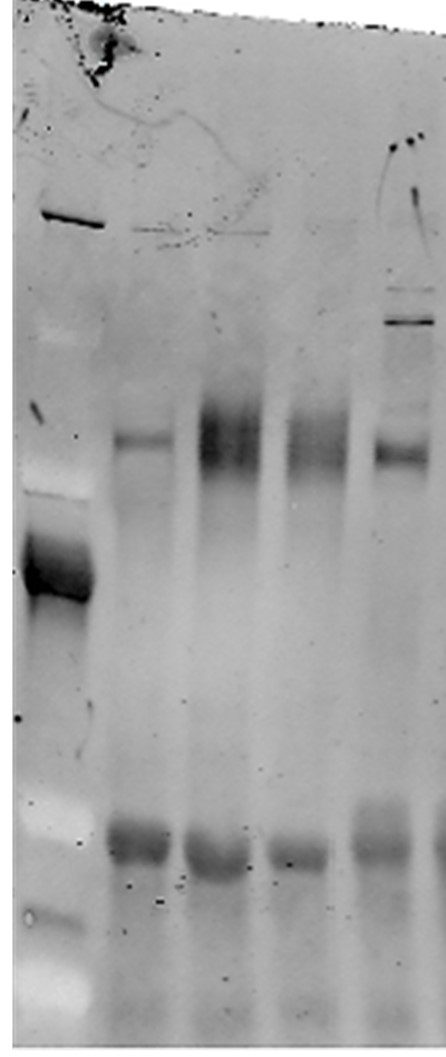

Supplement: Supplementary file 1 [file DataSheet_1.zip › beta1 integrin wb.jpg]

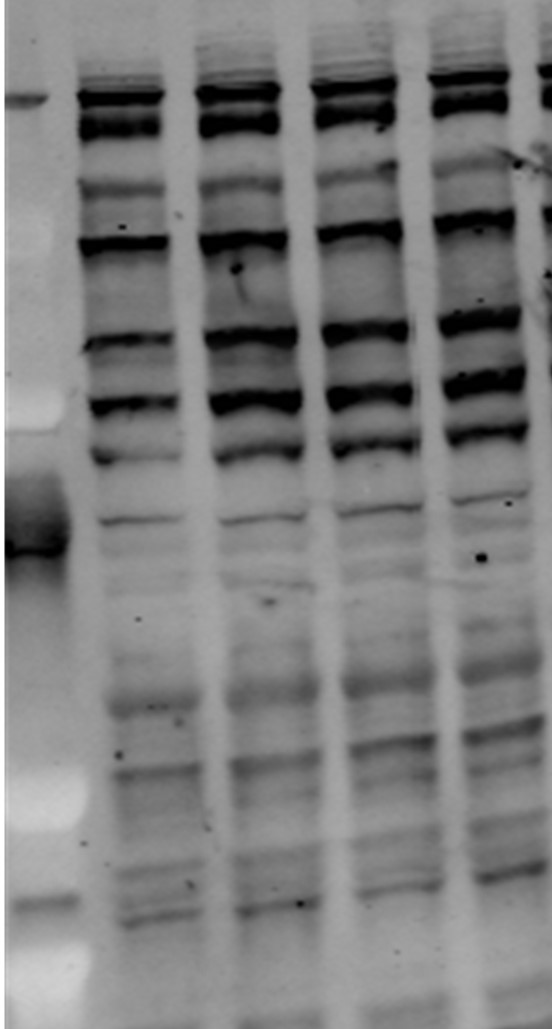

Supplement: Supplementary file 1 [file DataSheet_1.zip › beta1 integrin input.jpg]
